# Restarting Medications After Deprescribing in Adults Discharged From Hospital to Skilled Nursing

**DOI:** 10.1001/jamanetworkopen.2026.17264

**Published:** 2026-06-08

**Authors:** Thomas J. Reese, Sandra F. Simmons, Eduard E. Vasilevskis, Emily K. Hollingsworth, Matthew S. Shotwell, Amanda S. Mixon

**Affiliations:** 1 Department of Biomedical Informatics, Vanderbilt University Medical Center, Nashville, Tennessee; 2 Department of Medicine, Division of Geriatrics, Geriatric Research, Education and Clinical Center (GRECC), VA Tennessee Valley Healthcare System, Nashville; 3 Division of General Internal Medicine and Public Health, Geriatric Research, Education and Clinical Center (GRECC), VA Tennessee Valley Healthcare System, Nashville; 4 Center for Quality Aging, Vanderbilt University Medical Center, Nashville, Tennessee; 5 Biostatistics and Anesthesiology, Vanderbilt University Medical Center, Nashville, Tennessee; 6 Department of Medicine, Division of General Internal Medicine and Public Health, VA Tennessee Valley Healthcare System, Nashville

## Abstract

**Importance:**

Deprescribing interventions safely reduce polypharmacy in hospitalized older adults, but the durability of medication reductions and factors associated with medication restart are poorly understood.

**Objective:**

To characterize medication restart patterns after hospital-initiated deprescribing, identify factors associated with restart, and examine associations between restart and acute health care utilization.

**Design, Setting, and Participants:**

This cohort study analyzed data from 2 randomized trials: Best Possible Medication History, Evaluate, Deprescribing Recommendations, and Synthesis trial (conducted March 2016 to October 2020 at Vanderbilt University Medical Center and 22 affiliated postacute care facilities) and Veterans Affairs—Drug Reduction in Older Patients trial (conducted October 2019 to March 2023 at VA Tennessee Valley Healthcare System). Participants were aged 50 years or older, taking 5 or more prehospital medications, and discharged to a skilled nursing facility (SNF) for postacute care; analyses included all participants with 1 or more deprescribed medication. Data were analyzed from September 2024 to December 2025.

**Exposures:**

Trial interventions included comprehensive medication history, identification of deprescribing opportunities, participant or surrogate interview to assess agreement, and collaboration with clinicians. Patients in the control group received a comprehensive medication history followed by usual care, which included routine deprescribing by their practitioners.

**Main Outcomes and Measures:**

Medication restart (reinitiation or return to previous or higher dose) was determined from discharge documentation and structured patient or surrogate interviews at SNF discharge, 7 and 90 days after SNF discharge. The following were assessed: restart frequency, timing, and medication class; factors associated with restart; and associations between restart and emergency department visits and hospital readmissions.

**Results:**

Among 598 participants, the median (IQR) age was 74.0 (67.2-82.0) years, 354 (59.2%) were male, and 417 (69.7%) restarted 1 or more deprescribed medication. Of 8734 total deprescribed medications, 1385 (15.9%) were restarted across 1461 restart episodes; 330 (22.6%) occurred during the SNF stay, 423 (29.0%) between SNF discharge and 7-day follow-up, and 708 (48.5%) between 7- and 90-day follow-ups. Overall, approximately 84% of deprescribed medications remained discontinued at 90 days. Restart rates were similar between intervention and usual care groups. Higher health literacy and longer intervention duration were associated with lower restart risk, whereas higher baseline medications, more prescribers, and use of 1 to 2 (vs ≥3) pharmacies were associated with a higher risk. Restart during the SNF stay was associated with higher 90-day hospital readmission.

**Conclusions and Relevance:**

In this cohort study, approximately 1 in 6 deprescribed medications were restarted within 90 days, with nearly half occurring soon after SNF discharge. Patient factors and markers of care fragmentation were associated with restart, suggesting that enhancing transitional care and postdischarge support may improve the durability of hospital-initiated deprescribing.

## Key Points

**Question:**

Among older adults discharged to skilled nursing facilities (SNFs) after hospital-initiated deprescribing, what is the frequency and timing of medication restarts, what factors are associated with restarts, and is there an association between restarts and health care utilization?

**Findings:**

In this cohort study analyzing data from 2 randomized trials with a total of 598 participants, 15.9% of deprescribed medications were restarted. Higher health literacy and longer intervention exposure were associated with fewer restarts, whereas more prescribers, higher number of baseline medications, and fewer pharmacies were associated with medication restarts; restart during the SNF stay was associated with greater 90-day hospital readmissions.

**Meaning:**

These findings suggest deprescribing durability for hospitalized older patients declines after the SNF-to-home transition; targeting high-risk patients and improving prescriber-pharmacy coordination during care transitions may reduce medication restarts and hospital readmissions.

## Teaser

This cohort study evaluates medication restart patterns after hospital-initiated deprescribing, identifies factors associated with restart, and examines associations between restart and acute health care utilization.

## Introduction

Polypharmacy, commonly defined as the use of 5 or more medications, affects 39% to 65% of community-dwelling older adults in the US and is also prevalent among hospitalized patients, where 45% experience polypharmacy.^1,2,3,4,5^ Among hospitalized older adults, the prevalence of potentially inappropriate medications (PIMs) is high, with studies reporting that up to 70% receive at least 1 PIM during hospitalization.^6,7,8^ PIMs, medications for which the potential harms are likely to outweigh the potential benefits in older adults, are associated with approximately 2-fold higher risk of adverse drug event-related hospitalization, increased emergency department visits, more adverse drug events, and higher mortality.^6,9^

Deprescribing is the supervised medication withdrawal or dose reduction to minimize polypharmacy and medication-related harm.^10,11^ Randomized trials in hospital and postacute care settings show that deprescribing interventions can reduce overall medication burden and PIM use without increasing adverse drug events.^12,13^ The Best Possible Medication History, Evaluate, Deprescribing Recommendations, and Synthesis (Shed-MEDS) trial evaluated a patient-centered deprescribing intervention initiated during hospitalization for older adults transitioning to a skilled nursing facility (SNF), a postacute inpatient rehabilitation and nursing care setting. Results showed a 14% reduction in total medications at SNF discharge and a 15% reduction maintained 90 days after SNF discharge.^12^ Systematic reviews and meta-analyses have consistently shown that deprescribing interventions can be implemented safely using appropriate tapering protocols.^13,14^ In these studies, adverse drug withdrawal events accounted for 1.8% to 19.7% of all adverse drug events, and the frequency did not differ between deprescribing intervention and control groups.

Despite the demonstrated effectiveness of deprescribing interventions, less is known about the durability of these effects. Specifically, little is known about the extent to which medications are restarted after deprescribing, the patient and health care system factors associated with medication restart, or the clinical implications of reinitiating previously discontinued medications.^15^ Frequent medication restarts may signal inadequate patient education, poor care coordination, or unresolved symptoms requiring treatment.^15,16^ Transitions from hospital to SNF and then home are periods when medication changes occur frequently and may be reversed due to communication breakdowns, involvement of multiple prescribers, and limited health literacy.^17,18,19^ Identifying factors associated with medication restart can inform the design of more durable, safe, and sustainable deprescribing interventions during care transitions.^11,15,19^ We retrospectively pooled 2 deprescribing trials to (1) quantify medication restart frequency and timing during care transitions, (2) identify patient and system factors associated with restart, and (3) examine restart associations with ED visits and readmissions.

## Methods

### Study Design and Participants

We retrospectively pooled data from 2 randomized trials: Shed-MEDS (March 2016 to October 2020) at Vanderbilt University Medical Center and Veterans Affairs—Drug Reduction in Older Patients trial (VA-DROP) (October 2019 to March 2023) at VA Tennessee Valley Healthcare System.^5,12^ Both trials used the same eligibility criteria and patient-centered deprescribing protocol initiated during hospitalization, and both trials and the present study were approved by their institutional review boards. In the parent trials, all patients or surrogates provided written informed consent for their participation. We followed the Strengthening the Reporting of Observational Studies in Epidemiology (STROBE) reporting guidelines for cohort studies.^20^

Eligible participants were community-dwelling, aged 50 years or older, taking 5 or more prehospital medications, hospitalized at the study sites, and discharged to an SNF for postacute care. Exclusion criteria were non-English speaking, long-stay nursing home residents, or life expectancy less than 6 months. We included participants from both intervention and usual care groups with at least 1 medication deprescribed during the hospitalization or SNF stay, whether as part of the trial intervention or usual clinical care. We excluded vitamins and supplements because these agents are often over the counter and not routinely monitored by prescribers.

### Intervention

The deprescribing intervention protocol has been described previously.^5,21^ Briefly, research clinicians (pharmacists or nurse practitioners) conducted a comprehensive medication history, identified deprescribing opportunities based on patient- and medication-specific factors, and discussed recommendations with patients or surrogates to assess their agreement, followed by communicating recommendations with relevant prescribers.^21,22,23^ Deprescribing included stopped medications or dose reductions for scheduled and as-needed medications and was incorporated into hospital discharge orders when possible and communicated to the SNF care team within 48 hours of SNF admission. Control participants received a comprehensive medication history by the research team followed by usual care, wherein deprescribing and restarts could occur as part of routine clinical care. Participants were followed up through the SNF stay and for 90 days after SNF discharge.

### Measures and Outcomes

Baseline characteristics included age, sex, medication assistance, number of outpatient prescribers, number of pharmacies used in the previous 6 months, hospital duration (admission to discharge), intervention duration (trial enrollment to hospital discharge), number of medications at baseline (trial enrollment), and Brief Health Literacy Screen (BHLS) total score (scores 3-15, higher scores indicate greater health literacy). The BHLS has demonstrated internal consistency (Cronbach α = .80) and validity in clinical populations.^24,25^ Race and ethnicity were gathered from participants’ medical records and patients were asked to confirm via interview to ensure accuracy. Race and ethnicity were assessed as required by the parent trial funding.

Deprescribing included both complete discontinuation or dose reduction of existing medications, as documented in the parent trials, and applied to both scheduled and prescribed-as-needed (PRN) medications. Medication restart was defined as either reinitiation of a previously discontinued medication or escalation of the dose back to, or above, the predeprescribing dose. Neither trial independently documented switches between drug classes or by indication, although all newly prescribed medications were documented in both trials. We assessed restarts at 3 prespecified time points: SNF discharge and 7 and 90 days after SNF discharge. Missing data reflected loss to follow-up in the parent trials and were not imputed for this study. We used structured patient or surrogate interviews and review of medical record documentation to determine whether each deprescribed medication had been reinitiated since the prior time point for restart classification.

We described the frequency and types of restarted medications by pharmacologic class, restart timing across follow-up intervals, and compared restart rates between intervention and usual care groups. We examined associations between patient- and system-level factors (see Conceptual Framework section) and the rate of medication restart. We also evaluated whether medication restart was associated with a change in emergency department visits and hospital readmissions through 90-day follow-up; these acute health care utilization outcomes were ascertained from the health system electronic medical record, with events reported via patient or surrogate interviews reconciled to the record when applicable. The analysis focused on the occurrence and timing of restarts, not the clinical appropriateness of each restart, which was not documented in the parent trials.

### Conceptual Framework

We selected covariates based on a conceptual framework positing that medication restart after deprescribing is associated with 3 domains: patient characteristics, clinical-need factors, and health system or transition-of-care factors. Patient characteristics included age, sex, health literacy, baseline medication burden, and need for assistance with medications. Clinical-need factors such as symptom severity (eg, blood pressure or glucose control) and explicit clinician rationale for restart were not independently documented in the parent trials. Health system and transition-of-care factors included hospital length of stay, number of prescribers, number of pharmacies, and duration of exposure to the deprescribing intervention, which were treated as markers of care complexity and fragmentation. These domains informed our multivariable models to evaluate factors associated with medication restart.

### Statistical Analysis

We described participant characteristics stratified by medication restart status using median (IQR) for continuous variables and number (percentage) for categorical variables, with comparisons using Wilcoxon rank-sum and Pearson χ^2^ tests. To characterize medication restart patterns, we described the frequency and types of restarted medications by pharmacologic class and timing across follow-up intervals.

To compare restart rates between intervention and usual care groups, we fit mixed-effects logistic regression models with medication restart as the outcome and a random intercept for participant to account for repeated measures across study time points. Our primary model included fixed effects for assessment time point and randomization group, providing an overall effect of the deprescribing intervention on restart averaged across time. In a prespecified exploratory analysis, we additionally fit a model including a time-by-randomization group interaction and compared this with the primary model using a likelihood ratio test to assess heterogeneity of group differences over time.

To identify patient- and system-level factors associated with medication restart, we fit Poisson regression models with the count of medication restarts per participant over 90 days as the outcome, examining age, sex, BHLS score, medication assistance, number of prescribers and pharmacies, hospital and intervention length, and total baseline medications as independent variables; results are reported as rate ratios (RRs) with 95% CIs. Because emergency department visits and hospitalizations were conceptualized as downstream outcomes potentially associated with restart, these events were not included as factors in the restart models. To examine the association between medication restart and acute health care utilization following SNF discharge, we fit separate Poisson regression models with emergency department visits or hospitalizations as outcomes, adjusting for restart status at SNF and baseline covariates; results are reported as RRs with 95% CIs. Analyses followed intention-to-treat principles. Two-sided *P* < .05 was considered statistically significant. Data were managed using REDCap, and analyses were performed using R version 4.3.3 (R Project for Statistical Computing) from September 2024 to December 2025.

We examined restart patterns separately for reinitiation after complete discontinuation and dose escalations following dose reduction and repeated key analyses stratified by trial. In addition, we conducted a sensitivity analysis using modified Poisson regression with robust standard errors via generalized estimating equations to estimate relative risks for the binary outcome of any restart within 90 days.

## Results

### Participant Characteristics and Overall Restart Frequency

Table 1 shows baseline characteristics stratified by medication restart (yes or no) for the 598 total participants across both trials (intervention and control groups combined). A total of 354 (59.2%) were enrolled in Shed-MEDS and 244 (40.8%) were enrolled in VA-DROP. Participants across both trials had a median (IQR) age of 74.0 (67.2-82.0) years, 354 (59.2%) were male, 101 (16.9%) were Black, 486 (81.3%) were White, 16 (2.7%) were Hispanic, and 319 (53.3%) received medication assistance. Overall, 417 participants (69.7%) restarted 1 or more deprescribed medications. In unadjusted comparisons, those who restarted 1 or more deprescribed medications had shorter hospital stays, shorter intervention exposure, and higher rates of emergency department visits and hospitalizations than those who did not restart (Table 1).

**Table 1.** Participant Characteristics (N = 598)

Characteristic	Restarted 1 or more deprescribed medications, No. (%)		*P* value
	No (n = 181)	Yes (n = 417)	
Trial intervention	79 (43.6)	217 (52.0)	.07
Parent Trial Cohort			
Shed-MEDS	106 (58.6)	248 (59.5)	.91
VA-DROP	75 (41.4)	169 (40.5)	
Demographics			
Age, y, median (IQR)	72.9 (66.0-80.2)	74.7 (68.0-82.0)	.07
Sex			
Female	71 (39.2)	173 (41.5)	.67
Male	110 (60.8)	244 (58.5)	
Race			
Black	37 (20.4)	64 (15.3)	.04
White	140 (77.3)	346 (83.0)	
Other or declined to state^a^	2 (1.1)	2 (0.5)	
Ethnicity			
Hispanic	3 (1.7)	4 (1.0)	.35
Non-Hispanic	177 (97.8)	405 (97.1)	
Unknown, not reported, or declined	1 (0.6)	8 (1.9)	
Assistance with medications	96 (53.0)	223 (53.5)	.99
Health literacy, BHLS score (3-15)	11 (8-14)	12 (8-15)	.15
Health status			
Hospital length of stay, d, median (IQR)	8.9 (6.2-14.9)	7.7 (5.4-11.6)	.002
Length of trial intervention, d, median (IQR)	3.2 (1.2-7.1)	2.1 (1.0-5.0)	<.001
Medications			
No. of prescribers at baseline, median (IQR)	3 (2-4)	3 (2-4)	.79
No. of pharmacies used at baseline, median (IQR)	2 (1-2)	2 (1-2)	.27
No. of medications at baseline, median (IQR)	23 (19-28)	25 (20-29)	.05
No. of restarted medications	0	4 (2-6)	NA
Healthcare utilization			
Emergency department visit after SNF	3 (1.7)	41 (9.8)	<.001
Hospitalization after SNF	19 (10.5)	100 (24.0)	<.001

Abbreviations: BHLS, brief health literacy screen, NA, not applicable; Shed-MEDS, Best Possible Medication History, Evaluate, Deprescribing Recommendations, and Synthesis trial; SNF, skilled nursing facility; VA-DROP, Veterans Affairs—Drug Reduction in Older Patients trial.

^a^ Other included Asian and Native American or Alaska Native.

### Medication-Level Restart Patterns and Intervention vs Usual Care Comparisons

**Figure 1.**
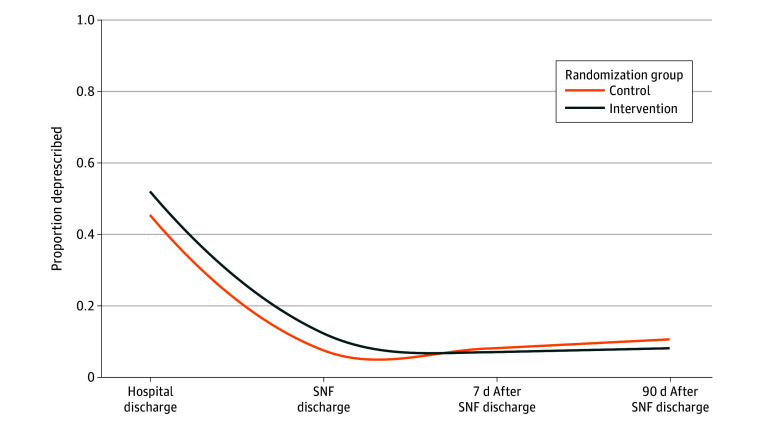
Line Chart Showing Proportion of Deprescribed Medications That Were Restarted Over Time Between Deprescribing Intervention and Control Groups SNF indicates skilled nursing facilities.

**Figure 2.**
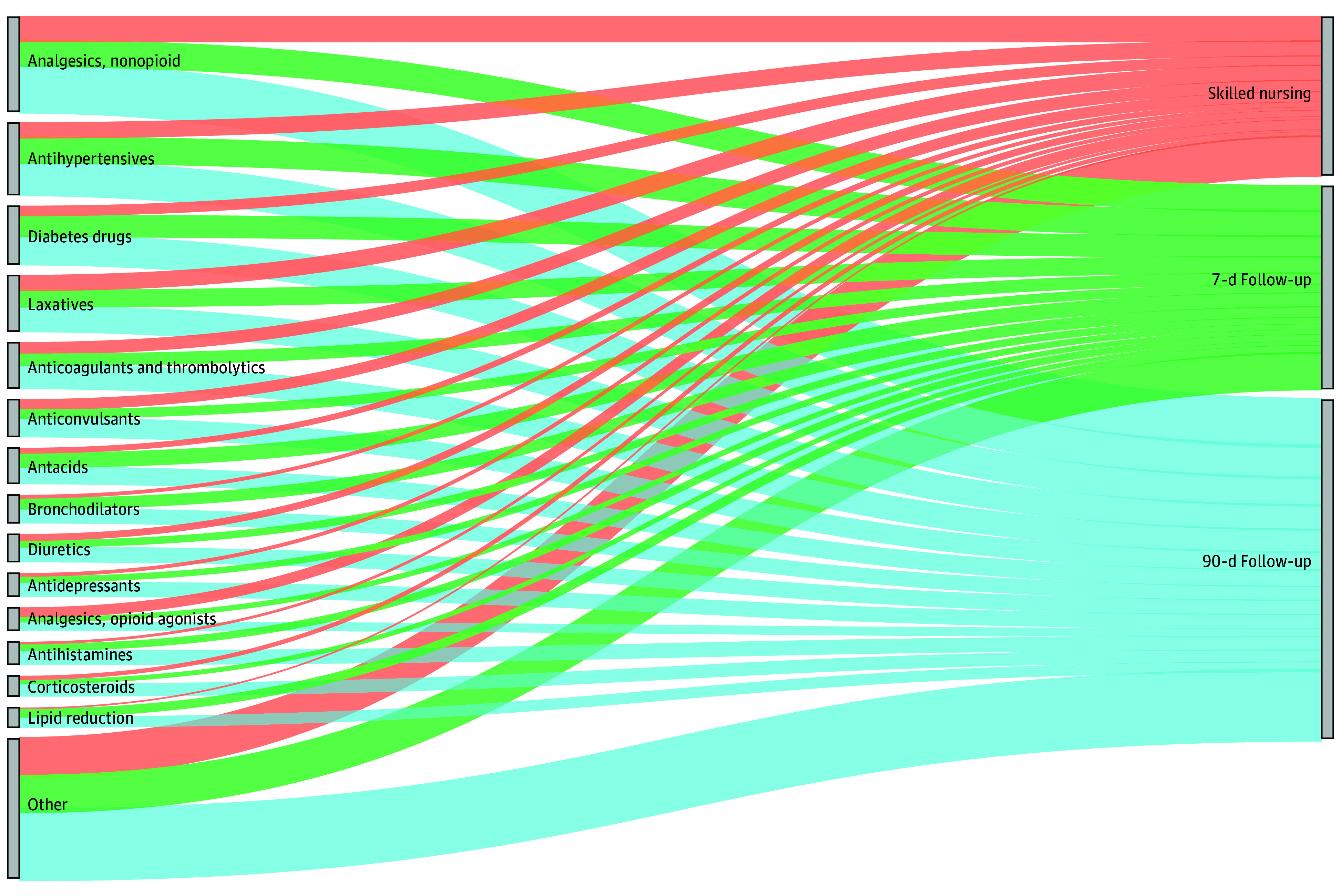
Alluvial Plot Showing Top 14 Medication Groups Restarted by Trial Periods

At the medication level, 1385 of 8734 deprescribed medications (15.9%) were restarted by 90 days after SNF discharge, representing 1461 total restart episodes. Nearly half of restart episodes occurred between 7 and 90 days after SNF discharge, and the daily restart rate was highest during the first week at home. Restart rates were similar between intervention and usual care groups across trials (Table 2, Figure 1); the overall RR for intervention vs usual care was 1.09 (95% CI, 0.91-1.31; *P* = .37), although an exploratory time-by-group interaction suggested some heterogeneity in restart probabilities over time (χ^2^ = 8.53; *P* = .014). The 3 most frequently restarted medication classes were nonopioid analgesics, antihypertensives, and diabetes medications (Figure 2).

**Table 2.** Medication Restart by Randomization Group and Trial

Study and group	Participants, No.	With ≥1 restart, No. (%)^a^	Deprescribed meds, No.	Restarted meds, No. (%)^a^	Restart episodes, No.
Shed-MEDS					
Control	180	120 (66.7)	3085	353 (14.8)	454
Intervention	174	128 (73.6)	3427	421 (16.1)	560
VA-DROP					
Control	122	80 (65.6)	2347	294 (16.2)	398
Intervention	122	89 (73.0)	2465	317 (16.5)	411
All					
Control	302	200 (66.2)	5432	647 (15.4)	852
Intervention	296	217 (73.3)	5892	738 (16.3)	971

Abbreviations: Shed-MEDS, Best Possible Medication History, Evaluate, Deprescribing Recommendations, and Synthesis trial; VA-DROP, Veterans Affairs—Drug Reduction in Older Patients trial.

^a^ Percentages are calculated as follows: participants with 1 or more restart = participants with 1 or more restarted medication ÷ all participants in that trial and group; restarted meds = restarted medications ÷ all deprescribed medications at the medication level.

### Factors Associated With Medication Restart

**Figure 3.**
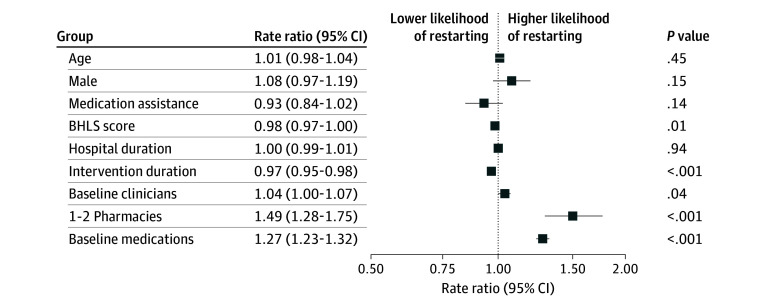
Dot Plot Showing Factors Associated With Restarting Medications Points are rate ratios (RR) from Poisson GLM; lines show 95% CIs. Dashed line indicates RR = 1. Age and baseline medications are expressed per 5 points. BHLS indicates brief health literacy screen.

In multivariable models, higher health literacy and longer intervention duration were associated with lower restart rates. Greater baseline medication burden, a higher number of prescribers, and use of 1 to 2 (vs ≥3) pharmacies were associated with higher restart rates (eTable 4 in Supplement 1, Figure 3).

### Restart and Health Care Utilization

Restart during the SNF stay was associated with higher 90-day hospital readmission (unadjusted RR, 1.17; 95% CI, 1.05-1.28; *P* = .002; adjusted RR, 1.19; 95% CI, 1.05-1.33; *P* = .004) compared with those who did not restart medications. In contrast, restart was not associated with the rate of emergency department visits (unadjusted RR, 1.09; 95% CI, 0.86-1.30; *P* = .42; adjusted RR, 1.08; 95% CI, 0.82-1.36; *P* = .54).

### Sensitivity Analyses

Age, sex, medication assistance, and hospital length of stay were not significantly associated with restart. Sensitivity analyses using a modified Poisson model for any restart within 90 days showed similar RRs in direction and magnitude (eTable 2 in Supplement 1). Results were similar when restarts after complete discontinuation were examined separately from dose increases (eTable 3 in Supplement 1). Across both trials, approximately one-quarter of deprescribed medications were later restarted after complete discontinuation and approximately 4% to 5% were restarted via dose increase, with comparable patterns by trial (eTable 4 in Supplement 1).

## Discussion

In 2 pooled hospital-initiated deprescribing trials for older adults transitioning to SNF for postacute care, nearly 70% of participants restarted 1 or more medications within 90 days of SNF discharge. However, approximately 30% maintained 1 or more deprescribed medications, and 84% of all deprescribed medications remained discontinued during the follow-up period. The deprescribing intervention in both trials did not change overall restart rates. A higher baseline medication burden, more prescribers, and the use of fewer pharmacies were factors associated with restarts, whereas higher health literacy and longer exposure to the deprescribing intervention were protective factors. Restart during the SNF stay was associated with higher 90-day hospital readmissions but not emergency department visits. The observed restart rates likely reflect both clinically appropriate responses to symptom recurrence or new diagnoses and potentially avoidable reversals of deprescribing decisions, which could not be distinguished within the available trial data.

These findings extend prior deprescribing intervention studies by evaluating the longer-term durability of deprescribing. The Shed-MEDS trial showed that deprescribing is feasible and safe but did not describe restart patterns, whereas this study combines 2 trial datasets to show that individual medications were frequently restarted even when total medication counts per participant remained lower following deprescribing.^12^ Although the associations between baseline factors and restart were statistically significant, the corresponding rate ratios were modest and should be interpreted as markers of restart risk at the population level rather than clinically decisive effects for individual patients. Together, these results indicate that while restarts are common at the patient and medication level, many patients maintain deprescribed regimens and most deprescribed medications remain stopped 90 days after the transition from SNF to home.

These findings are consistent with prior qualitative work showing that difficulty understanding medication changes, uncertainty about stopping medications, and concerns about symptom recurrence are key barriers to sustaining deprescribing; we found that higher health literacy and longer intervention duration were associated with fewer restarts, aligning with these themes.^22,26^ Prior work from the Shed-MEDS and VA-DROP has also shown that some older adults decline participation in deprescribing research because they feel overwhelmed by their current health status, mistrust research, or are hesitant to participate in a deprescribing study, which may contribute to medication restarts among those who do participate.^27^

System-level vulnerabilities during care transitions likely also contribute to restart.^17,28^ Prior studies^17,18,28^ describe high rates of medication discrepancies across hospital-SNF-home transitions, with discrepancies frequently involving chronic medications and contributing to adverse outcomes. Our finding that nearly half of restarts occurred between 7 and 90 days after SNF discharge, with the highest daily restart rate in the first week at home, underscores the SNF-to-home transition as a critical point for medication review and patient education.^28^ The association between more prescribers and higher restart rates is consistent with prior work^29,30^ showing that older adults seen by multiple prescribers and/or across multiple health care settings are more likely to experience polypharmacy and PIM use. Use of 1 to 2 pharmacies (vs ≥3 pharmacies) was unexpectedly associated with higher restart; in our data, pharmacy count reflects number of locations where patients reported filling prescriptions, and there was likely variation in prescriber-pharmacy communication and documentation not measured in this study.^31,32^ This finding is therefore hypothesis-generating rather than definitive evidence of fragmentation.

The association between restart and acute care utilization also aligns with Shed-MEDS trial findings, which showed no overall reduction in ED visits or readmissions.^33^ In the current study, restart during the SNF stay was associated with higher hospital readmissions, suggesting that restart may reflect clinical instability or complex treatment plans.^30,34^ It is also possible that frequent medication changes can contribute to patient confusion or nonadherence, or adverse drug events, thereby increasing hospitalization risk.^35,36,37^ Although causality cannot be inferred, these findings support targeting patients at high risk of restart for intensified postdischarge support in future work.^33,37^

These findings inform the broader deprescribing literature, where results consistently show short-term reductions in specific medications or PIMs but limited data on long-term sustainability, relapse, and restart patterns.^1,13,14,15,38,39^ Our study responds directly to calls to include more granular, medication-level outcomes and to capture health care utilization in deprescribing research.^38^ By identifying baseline medication burden, prescriber count, health literacy, and intervention exposure as factors associated with restart, this work informs potential measures for risk stratification and tailoring future deprescribing interventions, such as extending follow-up or enhancing transitional care for patients with high polypharmacy and multiple prescribers or those with lower health literacy.^2,34^

### Limitations

Trials were conducted within an academic medical center network and a Veterans Affairs health system in a single region, which may limit generalizability. Although the trials differed in some participant characteristics and medication restart patterns, adjustment for trial and trial-stratified analyses showed similar associations, supporting pooled analyses while acknowledging possible residual heterogeneity. The combined cohort was predominantly male and White, reflecting the underlying study populations. Medication use and restart were ascertained through structured interviews and available medical records, with potential misclassification, particularly for nonprescription or as-needed medications, although structured interviews explicitly asked about all medication types. Neither trial included independent documentation of the clinical appropriateness of individual medication restarts; thus, we could not distinguish between clinically appropriate restarts due to symptom recurrence or new diagnoses vs restarts that may have reflected patient uncertainty or communication gaps. Incomplete follow-up among trial participants may have contributed to an underestimate of restart and readmission rates if those at higher risk for these outcomes were more likely to be lost to follow-up. Additionally, the observational nature of this retrospective analysis precludes causal inference regarding the relationship between restart and readmission, and the follow-up period was limited to 90 days after SNF discharge.

## Conclusions

In this cohort study, medication restart after hospital-initiated deprescribing was common, especially after the SNF-to-home transition, and was associated with patient and system factors. In the context of prior trial findings and the broader deprescribing literature, these results suggest that effective deprescribing requires not only safe in-hospital medication reduction but also deliberate strategies to sustain those changes across care transitions and clinical settings, with focused support for patients at highest risk of restart.

## Notes

Supplement 1. eTable 1. Relative Risks for Any Medication Restart within 90 Days From Modified Poisson Regression With Robust Standard Errors Using Generalized Estimating Equations eTable 2. Rate Ratios for Counts of Restarts After Complete Discontinuation vs Dose Increases, by Participant eTable 3. Restart Type by Trial at Medication and Participant Levels eTable 4. Multivariable Associations Between Participant Factors and Medication Restart

Supplement 2. Data Sharing Statement

## References

[1] Linsky AM, Motala A, Booth M, Lawson E, Shekelle PG. Deprescribing in Community-Dwelling Older Adults: A Systematic Review And Meta-Analysis. JAMA Netw Open. 2025;8(5):e259375. doi:10.1001/jamanetworkopen.2025.9375 40338546 PMC12062908

[2] Pazan F, Wehling M. Polypharmacy in older adults: a narrative review of definitions, epidemiology and consequences. Eur Geriatr Med. 2021;12(3):443-452. doi:10.1007/s41999-021-00479-3 33694123 PMC8149355

[3] Charlesworth CJ, Smit E, Lee DSH, Alramadhan F, Odden MC. Polypharmacy among adults aged 65 years and older in the United States: 1988-2010. J Gerontol A Biol Sci Med Sci. 2015;70(8):989-995. doi:10.1093/gerona/glv013 PMC4573668 25733718

[4] Young EH, Pan S, Yap AG, Reveles KR, Bhakta K. Polypharmacy prevalence in older adults seen in United States physician offices from 2009 to 2016. PLoS One. 2021. doi:10.1371/journal.pone.0255642 PMC8330900 34343225

[5] Vasilevskis EE, Shah AS, Hollingsworth EK, ; Shed-MEDS Team. A patient-centered deprescribing intervention for hospitalized older patients with polypharmacy: rationale and design of the Shed-MEDS randomized controlled trial. BMC Health Serv Res. 2019;19(1):165. doi:10.1186/s12913-019-3995-3 30871561 PMC6416929

[6] Puig T, Leache L, González-Senac NM, ; MAPAC-MPC Network. Prevalence of potentially inappropriate medications and prescription dynamics in elderly hospitalized patients in Spain. BMC Geriatr. 2024;24(1):798. doi:10.1186/s12877-024-05308-3 39350081 PMC11443693

[7] Alshammari H, Al-Saeed E, Ahmed Z, Aslanpour Z. Reviewing potentially inappropriate medication in hospitalized patients over 65 using explicit criteria: a systematic literature review. Drug Healthc Patient Saf. 2021;13:183-210. doi:10.2147/DHPS.S303101 34764701 PMC8572741

[8] Panamsky L, Ford A, Srivastava S, Wijeratne DT. Deprescribing potentially inappropriate medications in a tertiary care centre in Ontario. Can J Gen Intern Med. 2019;14(3):30-35. doi:10.22374/cjgim.v14i3.364

[9] Lim J, Jeong S, Jang S, Jang S. Hospitalization and emergency department visits associated with potentially inappropriate medication in older adults: self-controlled case series analysis. Front Public Health. 2023;11:1080703. doi:10.3389/fpubh.2023.1080703 37469702 PMC10352109

[10] Reeve E, Gnjidic D, Long J, Hilmer S. A systematic review of the emerging definition of ‘deprescribing’ with network analysis: implications for future research and clinical practice. Br J Clin Pharmacol. 2015;80(6):1254-1268. doi:10.1111/bcp.12732 27006985 PMC4693477

[11] Crisafulli S, Poluzzi E, Lunghi C, . Deprescribing as a strategy for improving safety of medicines in older people: clinical and regulatory perspective. Frontiers in Drug Safety and Regulation. Frontiers Media SA. 2022;2. doi:10.3389/fdsfr.2022.1011701

[12] Vasilevskis EE, Shah AS, Hollingsworth EK, . Deprescribing medications among older adults from end of hospitalization through postacute care: a Shed-MEDS randomized clinical trial. JAMA Intern Med. 2023;183(3):223-231. doi:10.1001/jamainternmed.2022.6545 36745422 PMC9989899

[13] Hanlon JT, Gray SL. Deprescribing trials: a focus on adverse drug withdrawal events. J Am Geriatr Soc. 2022;70(9):2738-2741. doi:10.1111/jgs.17883 PMC9489612 35596673

[14] Jamieson H, Nishtala PS, Bergler HU, . Deprescribing anticholinergic and sedative drugs to reduce polypharmacy in frail older adults living in the community: a randomized controlled trial. J Gerontol A Biol Sci Med Sci. 2023;78(9):1692-1700. doi:10.1093/gerona/glac249 36692224 PMC10460556

[15] Thio SL, Nam J, van Driel ML, Dirven T, Blom JW. Effects of discontinuation of chronic medication in primary care: a systematic review of deprescribing trials. Br J Gen Pract. 2018;68(675):e663-e672. doi:10.3399/bjgp18X699041 30249607 PMC6145971

[16] Namikawa K, Björnsson ES. Rebound acid hypersecretion after withdrawal of long-term proton pump inhibitor (PPI) treatment—are PPIs addictive? Int J Mol Sci. 2024;25(10):5459. doi:10.3390/ijms25105459 38791497 PMC11122117

[17] Tjia J, Bonner A, Briesacher BA, McGee S, Terrill E, Miller K. Medication discrepancies upon hospital to skilled nursing facility transitions. J Gen Intern Med. 2009;24(5):630-635. doi:10.1007/s11606-009-0948-2 19291332 PMC2669872

[18] King BJ, Gilmore-Bykovskyi AL, Roiland RA, Polnaszek BE, Bowers BJ, Kind AJH. The consequences of poor communication during transitions from hospital to skilled nursing facility: a qualitative study. J Am Geriatr Soc. 2013;61(7):1095-1102. doi:10.1111/jgs.12328 23731003 PMC3714367

[19] Kerstenetzky L, Birschbach MJ, Beach KF, Hager DR, Kennelty KA. Improving medication information transfer between hospitals, skilled-nursing facilities, and long-term-care pharmacies for hospital discharge transitions of care: A targeted needs assessment using the Intervention Mapping framework. Res Social Adm Pharm. 2018;14(2):138-145. doi:10.1016/j.sapharm.2016.12.013 28455194 PMC5699964

[20] von Elm E, Altman DG, Egger M, Pocock SJ, Gøtzsche PC, Vandenbroucke JP; STROBE Initiative. The Strengthening the Reporting of Observational Studies in Epidemiology (STROBE) statement: guidelines for reporting observational studies. Bull World Health Organ. 2007;85(11):867-872. doi:10.2471/BLT.07.045120 18038077 PMC2636253

[21] Shah AS, Hollingsworth EK, Shotwell MS, Mixon AS, Simmons SF, Vasilevskis EE. Sources of medication omissions among hospitalized older adults with polypharmacy. J Am Geriatr Soc. 2022;70(4):1180-1189. doi:10.1111/jgs.17629 34967444 PMC8986578

[22] Kim JL, Lewallen KM, Hollingsworth EK, Shah AS, Simmons SF, Vasilevskis EE. Patient-reported barriers and enablers to deprescribing recommendations during a clinical trial (Shed-MEDS). Gerontologist. 2023;63(3):523-533. doi:10.1093/geront/gnac100 35881109 PMC10028229

[23] Mixon AS, Hollingsworth E, Strayer TE III, . Engagement of outpatient providers in a deprescribing trial for hospitalized older patients transitioning to post-acute care facilities. Health Lit Commun Open. 2025;3(1):2587314. doi:10.1080/28355245.2025.2587314 41550262 PMC12811018

[24] Wallston KA, Cawthon C, McNaughton CD, Rothman RL, Osborn CY, Kripalani S. Psychometric properties of the brief health literacy screen in clinical practice. J Gen Intern Med. 2014;29(1):119-126. doi:10.1007/s11606-013-2568-0 23918160 PMC3889960

[25] Chew LD, Bradley KA, Boyko EJ. Brief questions to identify patients with inadequate health literacy. Fam Med. 2004;36(8):588-594. 15343421

[26] Crutzen S, Baas G, Abou J, . Barriers and enablers of older patients to deprescribing of cardiometabolic medication: a focus group study. Front Pharmacol. 2020;11:1268. doi:10.3389/fphar.2020.01268 32973509 PMC7468428

[27] Strayer TE, Hollingsworth EK, Shah AS, Vasilevskis EE, Simmons SF, Mixon AS. Why do older adults decline participation in research? Results from two deprescribing clinical trials. Trials. 2023;24(1):456. doi:10.1186/s13063-023-07506-7 37464431 PMC10353211

[28] Vasilevskis EE, Trumbo SP, Shah AS, . Medication discrepancies among older hospitalized adults discharged from post-acute care facilities to home. J Am Med Dir Assoc. 2024;25(7):105017. doi:10.1016/j.jamda.2024.105017 38754476 PMC11335011

[29] Hagiwara S, Komiyama J, Iwagami M, . Polypharmacy and potentially inappropriate medications in older adults who use long-term care services: a cross-sectional study. BMC Geriatr. 2024;24(1):696. doi:10.1186/s12877-024-05296-4 39169279 PMC11337775

[30] Krustev T, Milushewa P, Tachkov K. Impact of polypharmacy, drug-related problems, and potentially inappropriate medications in geriatric patients and its implications for Bulgaria-narrative review and meta-analysis. Front Public Health. 2022;10:743138. doi:10.3389/fpubh.2022.743138 35309221 PMC8927684

[31] Dinh TS, Hanf M, Klein AA, . Informational continuity of medication management in transitions of care: Qualitative interviews with stakeholders from the HYPERION-TransCare study. PLoS One. 2024. doi:10.1371/journal.pone.0300047 PMC10996284 38573912

[32] Hambrook M, Peterson S, Gorman S, Becotte G, Burrows A. Medication management surrounding transitions of care: a qualitative assessment of community pharmacists’ preferences (MEMO TOC). Can Pharm J (Ott). 2020;153(5):301-307. doi:10.1177/1715163520947444 33110471 PMC7560558

[33] Lee JW, Hollingsworth EK, Shah AS, . Emergency department visits and hospital readmissions after a deprescribing intervention among hospitalized older adults. J Am Geriatr Soc. 2024;72(7):2038-2047. doi:10.1111/jgs.18945 38725307 PMC11226369

[34] Chang TI, Park H, Kim DW, . Polypharmacy, hospitalization, and mortality risk: a nationwide cohort study. Sci Rep. 2020;10(1):18964. doi:10.1038/s41598-020-75888-8 33144598 PMC7609640

[35] Boockvar K, Fishman E, Kyriacou CK, . Adverse events due to discontinuations in drug use and dose changes in patients transferred between acute and long-term care facilities. Arch Intern Med. 2004;164(5):545-550. doi:10.1001/archinte.164.5.545 15006832

[36] Mixon AS, Neal E, Bell S, Powers JS, Kripalani S. Care transitions: a leverage point for safe and effective medication use in older adults–a mini-review. Gerontology. 2015;61(1):32-40. doi:10.1159/000363765 25277280 PMC4479140

[37] Liaw S, Ragbir-Toolsie K, Kabir R, . Medication discrepancies across care transitions and the role of pharmacy technicians: a retrospective chart review. JAPhA Pract Innov. 2024;1(3):100009. doi:10.1016/j.japhpi.2024.100009

[38] Bayliss EA, Albers K, Gleason K, . Recommendations for outcome measurement for deprescribing intervention studies. J Am Geriatr Soc. 2022;70(9):2487-2497. doi:10.1111/jgs.17894 35648465 PMC9489620

[39] Ibrahim K, Cox NJ, Stevenson JM, Lim S, Fraser SDS, Roberts HC. A systematic review of the evidence for deprescribing interventions among older people living with frailty. BMC Geriatr. 2021;21(1):258. doi:10.1186/s12877-021-02208-8 33865310 PMC8052791

