# Supplementary material for: Restarting Medications After Deprescribing in Adults Discharged From Hospital to Skilled Nursing
Source: JAMA Netw Open. 2026 Jun 8;9(6):e2617264. doi: 10.1001/jamanetworkopen.2026.17264 (PMC13247801; doi:10.1001/jamanetworkopen.2026.17264)
Supplement: Supplement 1. — eTable 1. Relative Risks for Any Medication Restart within 90 Days From Modified Poisson Regression With Robust Standard Errors Using Generalized Estimating Equations eTable 2. Rate Ratios for Counts of Restarts After Complete Discontinuation vs Dose Increases, by Participant eTable 3. Restart Type by Trial at Medication and Participant Levels eTable 4. Multivariable Associations Between Participant Factors and Medication Restart [file jamanetwopen-e2617264-s001.pdf]

Supplemental Online Content

Reese TJ, Simmons SF, Vasilevskis EE, Hollingsworth EK, Shotwell MS, Mixon AS. Restarting medications after deprescribing. *JAMA Netw Open*. 2026;9(6):e2617264. doi:10.1001/jamanetworkopen.2026.17264

eTable 1. Relative Risks for Any Medication Restart within 90 Days From Modified Poisson Regression With Robust Standard Errors Using Generalized Estimating Equations

eTable 2. Rate Ratios for Counts of Restarts After Complete Discontinuation vs Dose Increases, by Participant

eTable 3. Restart Type by Trial at Medication and Participant Levels

eTable 4. Multivariable Associations Between Participant Factors and Medication Restart

This supplemental material has been provided by the authors to give readers additional information about their work.

© 2026 [First author last name and initials or group name] et al. [Journal name].

eTable 1. Relative Risks for Any Medication Restart within 90 Days From Modified Poisson Regression With Robust Standard Errors Using Generalized Estimating Equations

Factor	Risk ratio	95% CI	*P* value
Age, per year	1.01	1.00–1.01	0.069
Male (vs female)	1.08	0.96–1.22	0.189
BHLS score, per point	1.01	0.99–1.02	0.375
Medication assistance (Yes vs No)	1.03	0.92–1.15	0.566
Number of prescribers, per prescriber	0.99	0.95–1.03	0.590
Pharmacies (1–2 vs ≥3)	1.32	1.07–1.63	0.008
Hospital stay, per day	1.00	0.99–1.01	0.773
Intervention duration, per day	0.97	0.95–0.99	0.015
Baseline medications, per medication	1.01	1.00–1.02	0.010

© 2026 [First author last name and initials or group name] et al. [Journal name].

eTable 2. Rate Ratios for Counts of Restarts After Complete Discontinuation vs Dose Increases, by Participant

Model	Factor	Rate ratio	95% CI	P value
**Restart after complete stop**				
	Age, per year	1.00	1.00–1.01	0.171
	Male (vs female)	1.01	0.91–1.11	0.905
	BHLS score, per point	1.00	0.99–1.01	0.691
	Medication assistance (Yes vs No)	0.90	0.82–0.98	0.017
	Number of prescribers, per prescriber	0.99	0.96–1.02	0.419
	Pharmacies (1–2 vs ≥3)	1.22	1.08–1.39	0.002
	Hospital stay, per day	1.00	0.99–1.01	0.694
	Intervention duration, per day	0.96	0.95–0.97	<0.001
	Baseline medications, per medication	1.05	1.04–1.05	<0.001
	VA-DROP (vs Shed-MEDS)	1.09	0.98–1.22	0.096
**Restart due to dose increase**				
	Age, per year	1.00	0.99–1.01	0.507
	Male (vs female)	1.02	0.79–1.31	0.897
	BHLS score, per point	0.98	0.95–1.01	0.141
	Medication assistance (Yes vs No)	0.74	0.59–0.93	0.008
	Number of prescribers, per prescriber	1.03	0.96–1.11	0.376
	Pharmacies (1–2 vs ≥3)	1.53	1.11–2.17	0.012
	Hospital stay, per day	1.01	0.99–1.03	0.203
	Intervention duration, per day	0.96	0.93–0.99	0.011
	Baseline medications, per medication	1.04	1.02–1.05	<0.001
	VA-DROP (vs Shed-MEDS)	1.41	1.09–1.83	0.009

Rate ratios and 95% CIs are from Poisson regression models for counts of restarts over 90 days; models are fit separately for restarts after complete discontinuation and for restarts due to dose increase.

© 2026 [First author last name and initials or group name] et al. [Journal name].

eTable 3. Restart Type by Trial at Medication and Participant Levels

Level	Trial	No. of medications/participants	Restarts after stop, No. (%)	Restarts due to dose increase, No. (%)	Median restarts after stop per participant	Median restarts due to dose increase per participant
**Medication**	Shed-MEDS	5001	1406 (28.1)	199 (4.0)	–	–
**Medication**	VA-DROP	3733	974 (26.1)	183 (4.9)	–	–
**Participant**	Shed-MEDS	354	–	–	4	0
**Participant**	VA-DROP	244	–	–	3	0

© 2026 [First author last name and initials or group name] et al. [Journal name].

eTable 4. Multivariable Associations Between Participant Factors and Medication Restart

**Factor**	**Rate ratio for restart (95% CI)[a]**	***P* value**
Age, per year	1.00 (0.99-1.00)	.45
Male sex (vs female)	1.08 (1.03-1.13)	.15
BHLS score, per 1-point increase	0.98 (0.98-1.00)	.01
Medication assistance (yes vs no)	0.93 (0.88-0.98)	.14
No. of outpatient prescribers, per prescriber	1.04 (1.00-1.07)	.04
Pharmacies, 1-2 vs ≥3	1.49 (1.28-1.75)	<.001
Hospital length of stay, per d	1.00 (0.99-1.01)	.94
Intervention duration, per d	0.97 (0.95-0.98)	<.001
Baseline medication count, per medication	1.05 (1.04-1.05)	<.001

[a]Rate ratios and 95% CIs are from Poisson regression models with the count of medication restarts per participant over 90 days as the outcome; all listed covariates are included simultaneously in the model.

© 2026 [First author last name and initials or group name] et al. [Journal name].
