# Supplementary material for: Restarting Medications After Deprescribing in Adults Discharged From Hospital to Skilled Nursing
Source: JAMA Netw Open. 2026 Jun 8;9(6):e2617264. doi: 10.1001/jamanetworkopen.2026.17264 (PMC13247801; doi:10.1001/jamanetworkopen.2026.17264)
Supplement: Supplement 2. — Data Sharing Statement [file jamanetwopen-e2617264-s002.pdf]

Data Sharing Statement

Reese. Restarting Medications After Deprescribing. *JAMA Netw Open*. Published June 08, 2026. doi:10.1001/jamanetworkopen.2026.17264

Data
Data available: No
